# Research on Genotype Markers for Plant Height and Assisted Breeding of Key Sorghum Resources in China

**DOI:** 10.3390/genes15010083

**Published:** 2024-01-09

**Authors:** Yubin Wang, Na Lv, Feng Yin, Guoqi Duan, Hao Niu, Jianqiang Chu, Haisheng Yan, Lan Ju, Fangfang Fan, Xin Lv, Junai Ping

**Affiliations:** 1 Sorghum Research Institute, Shanxi Agricultural University, Jinzhong 030600, China; wyb-444@163.com (Y.W.); nkyglsnh@126.com (H.N.); m17703541896_2@163.com (J.C.); yanhaisheng@sxau.edu.cn (H.Y.); julan1989@163.com (L.J.); fanfangfang199009@163.com (F.F.); lvxing_0_2000@126.com (X.L.); 2 State Key Laboratory of Sustainable Dryland Agriculture (in Preparation), Taiyuan 030031, China; 3 College of Agriculture, Shanxi Agricultural University, Taigu 030801, China; 15034782926@163.com (N.L.); yf98060413@163.com (F.Y.); dgq0928@163.com (G.D.)

**Keywords:** sorghum, plant height gene, breeding resources, molecular markers

## Abstract

Dwarfing and the selection of optimal plant types constitute the primary focus of sorghum breeding. However, the lack of clarity regarding the gene types associated with plant height genes *Dw1-Dw4* in the primary breeding materials has led to increased plant heights in improved offspring of the same plant height type, resulting in unsatisfactory morphological traits. This study aimed to elucidate the gene types related to plant height in breeding materials, validate the regulatory mechanisms, and establish a material improvement system. The goal was to achieve molecular-marker-assisted dwarf breeding through the detection of plant height genes and the test cross verification of main Chinese sorghum materials. Using 38 main male sterile lines and 57 main restorer lines of grain sorghum as materials, three plant height genes were detected and classified. Ninety-five F_1_ generation hybrids of these materials, along with typical materials, were measured at the wax maturity stage. Test cross results demonstrated that the variation in *dw1-dw3* genes in the breeding materials significantly influenced the plant height of hybrid offspring. The main male sterile lines in Chinese sorghum predominantly exhibited the “three-dwarf” type of Kafir and its improved lines, characterized by the genotype (*Dw1-Dw2-dw3-dw4*). On the other hand, restorer lines mainly showcased the improved “two-dwarf” (*Dw1-Dw2-dw3-dw4*) genotype of the Kaoliang/Caudatum subspecies, along with the “three-dwarf” type of some Kafir and its improved lines. The test materials predominantly contained *dw3* genes, with relatively fewer *dw1* genes in the restorer lines. The primary restorer materials lacked the *dw2* gene, and *dw2* significantly influenced plant type. The increased plant height in improved offspring of the same plant height type material was attributed to differences in gene types. Therefore, the enhancement of plant height in breeding materials should prioritize the use of different methods in conjunction with *Dw1* and *Dw2* classification.

## 1. Introduction

Sorghum, the fifth most widely cultivated cereal crop globally, is a C4 grass grown for grain, feed, forage, sugar, and biofuel [1]. Breeding for dwarfing traits in sorghum is crucial, with four major dwarfing genes identified as *Dw1–Dw4* [2]. In the early stages of hybrid utilization in China, sorghum primarily exhibited a “one-dwarf” hybrid, reaching heights of 2.5–3 m, leading to serious lodging. Recognizing the need for dwarf hybrids (approximately 2 m in height) for efficient production, Niu Tiantang et al. advocated for sorghum dwarf breeding and introduced Chinese sorghum hybrids regulated by the “two-dwarf” genotype [3]. However, with the rapid mechanization of agricultural production, the “two-dwarf” hybrid proved inadequate for mechanized production [4].

Currently, most commercial grain sorghum male sterile lines are “three-dwarf,” indicating the presence of three of the four dwarfing mutations [5]. Due to variations in plant height gene loci among different types of materials, hybrids from dwarf parents often exhibit increased plant height. The unclear location of plant height genes in main breeding materials has become a significant bottleneck in “three-dwarf” sorghum breeding. The classification of plant height genes in sorghum’s main breeding materials holds crucial guidance for enhancing the efficiency of sorghum dwarf breeding and the accuracy of hybrid selection.

In the 1950s, Quinby and Karper identified four loci, *Dw1-Dw4*, controlling height by modifying internode length in sorghum [6]. With advancements in molecular biology, *Dw1*, *Dw2*, and *Dw3* genes have been cloned. *Dw1*, located on chromosome 9 [7], influences internodal cell count, reducing it upon mutation [8,9]. *Dw2*, located on chromosome 6, when mutated, significantly reduces internode length [4,10,11]. *Dw3*, situated on chromosome 7 [5], exhibits a direct duplication of 882 bp on the fifth exon, leading to internode shortening, although its mutation stability is questionable [12]. *Dw4*, the fourth typical plant height locus, is believed to be around 6.6 Mb on chromosome 6, but associated genes remain uncloned [13]. Single genes can reduce plant height by up to 50 cm, and although their effects are additive, the reduction diminishes with each added dwarfing gene [14].

Dwarf varieties effectively mitigate the risk of sorghum lodging and represent the primary sorghum type cultivated in China. A comprehensive understanding of sorghum dwarf breeding requires not only the study of individual genes or materials but also the detection and classification of main breeding materials. This, combined with sorghum breeders’ practice of classifying materials by subspecies, can unveil crucial mechanisms, guiding plant height breeding. This study utilized 38 main male sterile lines and 57 main restorer lines from grain sorghum breeding to detect, classify, and analyze their plant height genes’ regulatory mechanisms through test cross experiments and material subspecies types. The goal was to facilitate plant-height-gene-assisted sorghum dwarf breeding.

## 2. Materials and Methods

This study employed gene sequencing and phenotypic verification methods for experimentation. The classification method utilized Quinby and Karper’s five-level plant height categorization [14].

### 2.1. Selection of Materials

The selected materials comprised parent materials and original resources from major sorghum breeding research units in China. A total of 95 sorghum trunk breeding materials were chosen, including 38 male sterile lines and 57 restorer lines (refer to Table 1).

### 2.2. Plant Height Genetic Detection Methods

Genomic DNA was isolated from sorghum seedlings using the DNA isolation kit (Tiangen Biotech (Beijing, China) Co. LTD). Primers were designed based on the sequences of dwarf genes, *dw1*, *dw2*, and *dw3*. PCR amplification was performed in a 20 μL mixture containing PCR Mix 10 μL, primer (100 μmol·L^−1^) 0.5 μL, DNA 0.5 μL, and ddH_2_O 8.25 μL. The amplification protocol included: 94 °C for 2 min; 32 cycles of 94 °C for 30 s, 55 °C for 30 s, and 72 °C for 45 (120) s; and 72 °C for 10 min. PCR products of *dw1* and *dw2* were sent to Nanjing Plai Biotechnology Co Ltd. for sequencing, and subsequent sequence analysis was performed using DNAman v6 software. The PCR products of *dw3* were separated in a 1% agarose gel and visualized by ethidium bromide staining (refer to Table 2).

### 2.3. Phenotypic Verification Methods

The experiment was conducted in the winter of 2020 at the Shibu Farm in Sanya City, Hainan Province, China (109°44′ E, 18°29′ N), which has a tropical maritime monsoon climate. Diallel crosses were designed among typical male sterile lines A2V4A/B, SX44A/B, CS3541A/B, TX623A/B, and TX3197A/B, while test crosses were conducted between male sterile lines A2V4A, SX44A, TX623A, and TX3197A, and restorer lines SX1042, Jingliang 5, SRX0-30, and TX7078.

For the phenotypic analysis of the plant height assay in 2021, the test materials were planted at the Dongbai Experimental Base of the Sorghum Research Institute of Shanxi Agricultural University, Shanxi Province, China (37°35′ N, 112°42′ E). This location has a typical warm temperate semi-humid continental monsoon climate. Field plants were grown in double rows on soil with uniform nutrients. At the wax maturity stage, plant height, spike length, spike stalk length, stem height, and height under the flag leaf were measured (three times) for 95 accessions and all hybrid F_1_ generations.

### 2.4. Data Analysis Methods

The experiment was set up in three replications in a randomized complete block design (Jinzhong, Shanxi Province, China) on 5 m^2^ plots. At the wax maturity stage, after removing all leaves, plant height, spike length, spike stalk length (the length of the stem between the flag leaf ring and the bottom of the panicle), stem height, and height under the flag leaf (stem length—spike stalk length) were measured. Data for all measurements represent the average of three replicates. Mean value, standard deviation, and coefficient of variation were processed using Excel 2007 software.

## 3. Results

### 3.1. Detection of Three Plant Height Genes in Main Sorghum Breeding Materials

#### 3.1.1. Detection of dw1 Gene

The amplification length of *dw1* is 427 bp. At the position 1350 of the genome, nucleotide A changes to T, resulting in the *Dw1* mutation. When nucleotide position 1350 is A, the genotype is *dw1dw1*. With the change to T, the genotype becomes *Dw1Dw1* (refer to Supplementary Figure S1).

#### 3.1.2. Detection of dw2 Gene

The PCR product of *dw2* primer is 997 bp. Loss of GA at position 549 of the genome indicates the *dw2dw2* genotype. If it is not deleted, the genotype is *Dw2Dw2* (refer to Supplementary Figure S2).

#### 3.1.3. Detection of dw3 Gene

The *dw3dw3* amplification length is 1263 bp; when *dw3dw3* inserts 882 bp, mutating to *Dw3,* its amplification length becomes 2145 bp (refer to Supplementary Figure S3).

#### 3.1.4. Detection of dw4 Gene

The *Dw4* gene has not been cloned yet, but relevant scholars have detected it in broom sorghum [9]. Studies have shown that the *Dw4* genes of the grain sorghum Guinea Kafir were all recessive in the early stages of introduction. The genotype of *Dw4* in all materials in this experiment was *dw4dw4*.

### 3.2. Plant Height Gene Types of Main Breeding Materials

#### 3.2.1. “Three-Dwarf” Type

This type contains three gene types. *Dw1* is dominant (*Dw1Dw1-dw2dw2-dw3dw3-dw4dw4*), including 3 male sterile lines and 1 restorer line, with an average plant height of 110.58 cm. *Dw2* is the dominant genotype (*dw1dw1-Dw2Dw2-dw3dw3-dw4dw4*), and it is the primary type of the “three-dwarf” series, including 29 male sterile lines and 17 restorer lines, with an average plant height of 106.56 cm. *Dw3* is dominant (*dw1dw1-dw2dw2-Dw3Dw3-dw4dw4*), and there is only one accession of this genotype, NJ426, with a plant height of 90.33 cm (refer to Table 3).

#### 3.2.2. “Two-Dwarf” Type

This type contains three gene types. *Dw2* and *Dw3* are dominant genotypes (*dw1dw1-Dw2Dw2-Dw3Dw3-dw4dw4*), including three copies, all of which are male sterile lines, with an average plant height of 103.56 cm. *Dw1* and *Dw3* are the dominant genotype (*Dw1Dw1-dw2dw2-Dw3Dw3-dw4dw4*), and there is only 1 restorer line, Hegari, with a plant height of 217.00 cm. The dominant genotypes of *Dw1* and *Dw2* (*Dw1Dw1-Dw2Dw2-dw3dw3-dw4dw4*) are the main types of the “two-dwarf” gene type, containing a total of 30 materials, including 3 male sterile lines and 27 restorer lines, with an average plant height of 124.00 cm (refer to Table 3).

#### 3.2.3. “One-Dwarf” Type

This type has only one genotype. *Dw1*, *Dw2*, and *Dw3* are dominant genotypes (*Dw1Dw1-Dw2Dw2-Dw3Dw3-dw4dw4*). The 10 materials included are all restorer lines, with an average plant height of 179.53 cm. Since all test materials are homozygous, their genotypes are abbreviated from (*Dw1Dw1-Dw2Dw2-Dw3Dw3-dw4dw4*) to (*Dw1-Dw2-Dw3-dw4*), the same as below.

These results indicate that as the number of dwarf genes increases, the average plant height of the test materials gradually decreases from “one-dwarf” to “two-dwarf” to “three-dwarf” plant height types (refer to Table 3).

### 3.3. Plant Height Genotype and Subfamily Analysis of Main Breeding Materials

In 1972, Harlan and de Wet provided a simple classification of cultivated sorghum [15], categorizing them as (1) Bicolor, (2) Guineensia, (3) Caudatum, (4) Kafir, and (5) Durra. Subsequently, NI Vavilov and Chinese breeders Wang Defu et al. classified Chinese sorghum (Kaoliang) as a separate family [16]. Consequently, Chinese sorghum breeders typically categorize breeding materials into five classes: (1) Guineensia, (2) Caudatum, (3) Kafir, (4) Durra, and (5) Chinese sorghum (Kaoliang). In this study, we adopted this classification method for the test materials to provide better guidance for sorghum breeding.

Three of the four materials with dominant *Dw1* as the “three-dwarf” genotype belonged to the Durra family. The “three-dwarf” series of materials predominantly featured *Dw2* as the dominant genotype. With the exception of SX861, most of the included materials were Kafir or improvements of the Kafir family, with male sterile lines constituting the majority. The “two-dwarf” series of materials were primarily dominated by *Dw1* and *Dw2* as dominant genotypes. The included material subfamilies were mainly Chinese sorghum and the top group combination (Kaoliang/Caudatum), with the majority being restorer lines. Most of the “one-dwarf” types were Chinese sorghum (Kaoliang) and some improved varieties of Chinese sorghum, all of which were restorer lines, mainly comprising Chinese local varieties (refer to Table 3).

### 3.4. Analysis of the Distribution and Regulation of Plant Height Genes

Based on the survey, the spike length of the main genotypes showed a small difference ranging between 24.68 and 27.91 cm. The primary factor influencing sorghum plant height is the stem height. The majority of “three-dwarf” types exhibited genotypes where *Dw2* or *Dw3* is dominant. The average plant heights for these two genotypes were 110.58 and 106.56 cm, with average spike lengths of 25.67 and 27.91 cm, and average spike stalk lengths of 35.25 and 39.20 cm. The average height under the flag leaf was 49.67 and 39.45 cm. The primary genotype of the “two-dwarf” type was the one where *Dw1* and *Dw2* are both dominant. This genotype had an average plant height of 124.00 cm, average spike length of 31.80 cm, average spike stalk length of 26.38 cm, and average height under the flag leaf of 65.82 cm. The “one-dwarf” genotype was characterized by *Dw1*, *Dw2*, and *Dw3* all being dominant, with an average plant height of 179.53 cm, average spike length of 33.04 cm, average stalk length of 24.68 cm, and average height under the flag leaf of 121.82 cm. As we move from “three-dwarf” to “two-dwarf” to “one-dwarf” types, the average plant height gradually increases, while the average spike stalk length decreases. Therefore, the reduction of plant height under the flag leaf is the main reason leading to dwarf sorghum materials.

In the “three-dwarf” type, the height under the flag leaf of the dominant genotype *Dw3* was significantly higher than that of the dominant genotype *Dw2*. Therefore, the main regulatory genes for the dwarfing of plant height under the flag leaf in the “three-dwarf” type were *dw1* and *dw3.* This type predominantly included male sterile lines and a small number of restorer lines, mainly from the Kafir subspecies and its improved lines. In the “two-dwarf” type, the primary regulatory gene for plant height under the flag leaf was *dw3*. This type of sorghum was mainly comprised of restorer lines belonging to the Kaoliang/Caudatum subspecies combination (refer to Table 3).

Among the main breeding materials, the male sterile lines containing *dw2* were the “three-dwarf” types SX44B, SX4244B, and A2V4B, and restorer lines included NJ426, 1602N, and the “two-dwarf”-type restorer line Hegari. Three out of the six male sterile lines were of the Durra subspecies, and restorer line NJ426 is also an improved line of the Durra subspecies. Hegari and 1602N belong to the top subspecies (Caudatum) and its improved lines. Within the “three-dwarf” types, the ratio of spike stalk length to stem height in the genotype with (*Dw1-dw2-dw3-dw4*) was 0.43, lower than the 0.51 in the genotype with (*dw1-Dw2-dw3-dw4*). The ratio of spike stalk length to stem height in Hegari was 0.27, which is lower than the 0.34 in the dominant gene type of *Dw2* and *Dw3*. It is possible that the male sterile line subspecies Durra and the restorer line Caudatum subspecies contain the *dw2* allele, and this gene may regulate the ratio of spike stalk length to stem height (refer to Table 3).

### 3.5. Verification Analysis of the Main Plant Height Gene

In this study, two genotypes of the “three-dwarf” types were selected for test cross, specifically A2V4A/B and SX44A/B, both characterized by the (*Dw1-dw2-dw3-dw4*) genotype. Both A2V4A/B and SX44A/B belong to the Durra subspecies and serve as male sterile lines in the main breeding varieties. The genotypes of CS3541A/B, TX623A/B, and TX3197A/B are (*dw1-Dw2-dw3-dw4*), representing Durra, Kafir/Caudatum, and Kafir subspecies, respectively (refer to Table 4).

All five materials fall into the “three-dwarf” type, with slight differences in plant heights ranging from 126.33 to 115.01 cm. The results indicate that the two-way test cross plant heights of A2V4A and the same genotype SX44A were 132.60 and 130.26 cm, respectively. In contrast, the test cross plant heights of CS3541B, TX623B, and TX3197B with different genotypes were 220.11, 206.67, and 181.00 cm, respectively. The reverse test cross plant heights were 217.00, 200.01, and 175.12 cm, respectively. Notably, when the test cross progeny between A2V4A/SX44A and other materials all contained the *dw2* gene, the ratio of spike stalk length to stem height ranged between 0.3 and 0.38. However, the ratio of spike stalk length to stem height of the F_1_ generation without the *dw2* gene was only 0.35 for TX623A × TX3197B, while other F_1_ generations were ≥0.42, significantly higher than those containing the *dw2* gene. Therefore, the ratio of spike stalk length to stem height containing the *dw2* gene may be lower (refer to Table 4).

### 3.6. Validation Analysis of Sorghum Hybrid Combinations

To conduct test cross experiments, four male sterile lines with minor phenotypic differences in plant height and four restorer lines with slight phenotypic variations in plant height were carefully chosen. The primary genotypes of the restorer lines consisted of two “three-dwarf” types (*dw1-Dw2-dw3-dw4*) and two “two-dwarf” types (*Dw1-Dw2-dw3-dw4*); namely, TX7078, SX1042, Jinliang 5, and SXR0-30. Their respective plant heights were 112.67, 107.00, 138.00, and 124.67 cm.

The male sterile lines predominantly exhibited two genotypes of the “three-dwarf” types (*Dw1-dw2-dw3-dw4*) and (*dw1-Dw2-dw3-dw4*). Notably, A2V4A, SX44A, TX623A, and TX3197A showcased similar plant heights (126.33, 115.12, 128.12, and 115.01 cm, respectively). Test cross results revealed that the four combined genotypes tested with TX7078 and SX1042 as male parents, paired with A2V4A and SX44A, exhibited a genotype of (*Dw1dw1-Dw2dw2-dw3dw3-dw4dw4*) and an average plant height of 185.24 cm. Conversely, TX7078 and SX1042, in conjunction with TX623A, and the test crosses with TX3197A, demonstrated a genotype of (*Dw1Dw1-Dw2dw2-dw3dw3-dw4dw4*). Although the genotype of all these crosses was of the “two-dwarf” type, which possesses dominant *Dw1* and *Dw2* genes, the *dw1* loci in the second combination were *Dw1* homozygous genotype, resulting in an increased average plant height of 22.37 to 207.61 cm.

Moreover, the average plant height of the “three-dwarf” type with TX623A and TX3197A as female parents was (*dw1dw1-Dw2Dw2-dw3dw3-dw4dw4*). The combined genotype transformed to (*Dw1dw1-Dw2Dw2-dw3dw3-dw4dw4*) due to *dw1dw1* turning dominant. *Dw1dw1* became a “two-dwarf” type, with an average plant height of 194.33 cm, indicating an increase of 67.86 cm. These test cross results underscore that even with breeding materials of the same plant height type, genotype differences can lead to substantial variations in plant height among hybrid offspring. The homozygous plant height gene exerts a significantly stronger influence on regulating plant height than the heterozygous plant height gene (refer to Table 5).

## 4. Discussion

### 4.1. Enhancement of Plant Height Genotype Classification in Sorghum Breeding Materials

The inception of the first “green revolution” in agricultural production marked a significant milestone achieved by cultivating new lodging-resistant crop varieties with dwarf plants, substantially augmenting grain yields [17,18]. While China’s dwarf sorghum breeding originated with Jinza 5 [3], the current plant height genotypes of the primary breeding materials remain unclear. This study posits that the predominant sorghum main male sterile lines are primarily of the “three-dwarf” type, with a smaller subset being the “two-dwarf” type (refer to Table 3). Within the “three-dwarf” type, the majority of genotypes exhibit *Dw2* dominance (*dw1-Dw2-dw3-dw4*), while some genotypes showcase *Dw1* as dominant (*Dw1-dw2-dw3-dw4)* (refer to Table 3). Due to these genotypic variations, male sterile lines of the same “three-dwarf” type and similar plant height may produce offspring with increased plant height, potentially belonging to the “two-dwarf” type (refer to Table 4). Our findings reveal that materials with the genotype (*Dw1-dw2-dw3-dw4*) belong to the Durra subspecies, whereas those with the genotype (*Dw1-Dw2-dw3-dw4*) are predominantly from the Kafir subspecies and its improved lines. The Durra subspecies may carry the *dw2* allele, presenting an opportunity to enhance the genetic diversity of male sterile lines. Given that the majority of male sterile lines encompass *dw3* and *dw4* genes, it is advisable to classify these materials based on *Dw1* and *Dw2*. The utilization of molecular-assisted detection for *Dw1* and *Dw2* genes during offspring selection, particularly when working with Durra types, can expedite the selection process. This approach helps prevent issues such as the emergence of excessively tall offspring, thereby optimizing breeding efficiency.

The primary genetic types of restorer lines encompass “two-dwarf” (*Dw1-Dw2-dw3-dw4*) and “three-dwarf” types (*Dw1-Dw2-dw3-dw4*), with a minority exhibiting the “three-dwarf” type (*Dw1-Dw2-Dw3-dw4*) genotype. Originating from different sources, “two-dwarf” type restorer lines are mainly derived from the Kafir subspecies and its improved lines. In contrast, “three-dwarf” type restorer lines predominantly hail from the Kaoliang/Caudatum subspecies. The prevalent origin reflects Chinese breeders’ efforts to enhance Chinese local varieties for brewing sorghum. However, the plant height type of restorer lines is primarily “two-dwarf” (28 accessions), containing minimal *dw2* genetic material (refer to Table 3). When crossbred with male sterile lines of the “three-dwarf” type, their F_1_ progeny may assume the “two-dwarf” type, leading to increased plant height. This study advocates for improving restorer lines in tandem with male sterile lines, gradually transitioning toward the “three-dwarf” type while preserving the excellent traits of Chinese local varieties and enriching the source of improved materials. For instance, Hegari, which contains *dw2* in the plant height gene, can be strategically employed, aligning with male sterile lines classified based on *Dw1* and *Dw2*.

### 4.2. Primary Mechanisms of Gene Regulation in Sorghum Strains

Achieving a delicate balance between reducing stem length to prevent lodging and maintaining yield potential by not excessively reducing biomass has prompted the pursuit of breeding tall dwarfs [18,19,20,21,22]. Quinby and Karper’s identification of four loci, *Dw1-Dw4*, governing height by modifying internode length has been crucial in this endeavor [6]. Recessive alleles at these loci contribute to reduced internode length [6]. Pleiotropic effects of *Dw2* and *Dw3* encompass spike length, seed weight, and leaf area for *Dw2* [23,24], and seed weight, panicle size, tiller number, and leaf angle for *Dw3* [24,25,26]. Previous studies proposed that *dw1* and *dw3* act synergistically to reduce internode length [9].

Our findings reveal that the current trunk male sterile lines generally exhibit low plant height, all below 120 cm (Table 3). As we transition from “three-dwarf” to “two-dwarf” and “one-dwarf” types, the average plant height gradually increases, while the average spike stalk length decreases. Both “two-dwarf” and “one-dwarf” types are notably shorter than “three-dwarf” types. Therefore, reducing plant height under the flag leaves emerges as the primary criterion for selecting dwarf sorghum materials. In line with prior research, except for the *dw4* gene, present in all materials, *dw1* and *dw3* play regulatory roles in “three-dwarf” types, with *dw3* primarily regulating “two-dwarf” types [25]. Notably, Chinese sorghum breeders have predominantly eliminated the dominant *Dw3* gene during “two-dwarf” type improvement, focusing on adding the *dw1* gene and a small portion of the *dw2* gene in “three-dwarf” type enhancement (Table 3).

Research by Graham and Lessman proposed that mutations in plant height genes significantly reduce stem length, spike length, and seed weight without diminishing leaf number [23]. Our study indicates a scarcity of materials containing the *dw2* gene in main breeding materials. Josie L. Hilley posited that *dw2* mutations lead to a substantial reduction in inter-stem length, a trait selected in sorghum breeding programs [8]. Joel Oliver demonstrated that *dw2* mutations do not alter cell proliferation in new internodes in the apical dome but inhibit cell proliferation in extended internodes. This suggests *dw2′*s role in regulating cell proliferation during elongation, a crucial factor affecting internode length, plant height, light competition, and resource allocation to stem growth (sink strength) [27]. Our study contends that *dw1* and *dw3* solely shorten internode length without reducing node number, resulting in a larger spike stalk length ratio in dwarf materials. This causes severe leaf occlusion, impacting canopy structure and reducing light transmittance (Table 4).

Olson, S. N. et al. proposed that plant height is a key agronomic trait for regulating sorghum plant type, emphasizing genetic improvement of plant height as the main focus of sorghum dwarf breeding [28]. However, crop source strength depends on canopy photosynthetic characteristics and the canopy’s photosynthetic ability [29]. This could be a key reason for the limited breakthroughs in sorghum dwarf breeding. Josie L. Hilley asserted that *Dw2* influences internode length during the vegetative phase and the last 6–7 internodes produced after floral initiation [8]. Materials containing the *dw2* gene exhibit a smaller spike stalk length to stem height ratio, resulting in a more balanced plant structure. Therefore, the *dw2* gene should be enriched in material improvement efforts.

### 4.3. Additional Genes Influencing Plant Height in Sorghum

Gai Junyi introduced a genetic analysis method that considers genes with significant effects on quantitative traits as major genes and those with minor effects as polygenes. This approach enables the identification of major and polygenic effects, providing a more accurate and effective analysis of genetic impacts [30]. Sorghum plant height is governed by multiple genes. Existing research on sorghum plant height has primarily focused on *dw1*, *dw2*, and *dw3* genes, with limited exploration of *dw4*. Quinby and Karper suggested that variations in plant height within the same genotype result from the presence of a modification complex and minor genes, particularly influencing spike stalk length (the distance between the flag leaf ring and the base of the panicle) and spike length [6]. Even within populations with the same number of dwarf genes, significant height differences exist [6].

Our study demonstrates that as the dominance of plant height genes increases, the average plant height gradually rises, with substantial variations in plant height among materials of the same type. For instance, Hegari in the “two-dwarf” type reaches a height of 217.0 cm, significantly surpassing most other “two-dwarf” materials. Similarly, within the same gene type, such as the three-tall materials with the (*Dw1-Dw2-Dw3-dw4*) genotype, LN8RN, K35-Y5*1383, JR107, and SCS exhibit plant heights of 128.33, 101.33, 120.67, and 138.00 cm, respectively— noticeably lower than other materials (Table 3). Therefore, this study posits that aside from the *Dw4* gene eliminated by breeders in the early stages, other plant height genes exist [8,31].

In this study, the ratio of spike stalk length to stem height in different plant height gene types reveals a correlation between plant structure and plant height genes. Hence, breeders should not only focus on main genes but also explore those regulating spike stalk length and reducing node number to enhance sorghum plant morphology.

## 5. Conclusions

The primary male sterile lines for sorghum consist mainly of “three-dwarf” types from the Kafir subspecies and its improved lines, characterized by the (*Dw1-Dw2-dw3-dw4*) genotype. Restorer lines predominantly belong to the “two-dwarf” genotype (*Dw1-Dw2-dw3-dw4*) of the Kaoliang/Caudatum subspecies, along with some “three-dwarf” types from the Kafir subspecies and its improved lines. The test materials exhibit a higher prevalence of *dw3* genes, while restorer lines show relatively fewer *dw1* genes. Notably, the primary restorer lines lack the *dw2* gene, which influences plant structure. The observed variations in the plant height of improved offspring of the same type result from differences in genotype. Future efforts to enhance plant height in breeding materials should employ a combination of methods, focusing on the classification of *Dw1* and *Dw2*.

## Figures and Tables

**Table 1:** Subspecies classification and source of test materials.

Type	Number	Name	Subspecies	Source	Type	Number	Name	Subspecies	Source
**Male sterile line**	1	SX44B	Durra	China,SXAU,SRI	**Restorer line**	10	91644(H)	Kafir	Australia
	2	SX4244B	Durra	China,SXAU,SRI		11	91645(H)	Kafir	Australia
	3	A2V4B	Durra	China,SXAU,SRI		12	91648	Kafir	Australia
	4	CS3541B	Durra	China,SXAU,SRI		13	961547	Kafir	USA
	5	961542B	Durra	China,SXAU,SRI		14	91624(H)	Kafir	Australia
	6	F4B	Durra	China,SXAU,SRI		15	TX414	Kafir/Durra	USA
	7	TX3197B	Kafir	USA		16	NJ426	Kafir/Durra	China,SXAU,SRI
	8	(TXbmr6B/7501B)B	Kafir	China,SXAU,SRI		17	SX1042	Kaoliang/Kafir	China,SXAU,SRI
	9	N1B	Kafir	China,SXAU,SRI		18	HBNR436-2	Kaoliang/Kafir	China,HBAAS
	10	N3B	Kafir	China,SXAU,SRI		19	Hong yin zi	Kaoliang	China,Farmer kind
	11	ZSB	Kafir	China,SXAU,SRI		20	QKY	Kaoliang	China,Farmer kind
	12	TX414B	Kafir	USA		21	chf5933	Kaoliang	China,CFAI
	13	TX639B	Kafir	USA		22	SCS	Kaoliang	China,Farmer kind
	14	TX649B	Kafir	USA		23	20131937	Kaoliang	China,SXAU,SRI
	15	7501B	Kafir	USA		24	XL7	Kaoliang/Caudatu	China,XZAI
	16	TX2925B	Kafir	USA		25	JL5	Kaoliang/Caudatu	China,SXAU,SRI
	17	J16VII18B	Kafir	China,JLAAS		26	0-30/DHS	Kaoliang/Caudatu	China,SXAU,SRI
	18	J16VII27B	Kafir	China,JLAAS		27	SXR0-30	Kaoliang/Caudatu	China,SXAU,SRI
	19	SX605B	Kafir	China,SXAU,SRI		28	LNR	Kaoliang/Caudatum	China,LNAAS
	20	SX77B	Kafir	China,SXAU,SRI		29	XLH*GN2	Kaoliang/Caudatum	China,SXAU,SRI
	21	998B	Kafir	China,SXAU,SRI		30	0592F	Kaoliang/Caudatum	China,SXAU,SRI
	22	TV33B	Kafir	China,SXAU,SRI		31	SX861	Kaoliang/Caudatum	China,SXAU,SRI
	23	TX623B	Kafir/Caudatum	USA		32	1602N	Kaoliang/Caudatum	China,SXAU,SRI
	24	(314B/623B)B	Kafir/Caudatum	China,SXAU,SRI		33	HC356	Kaoliang/Caudatum	China,SXAU,SRI
	25	2055B	Kafir/Durra	China,JLAAS		34	1383-2	Kaoliang/Caudatum	China,SXAU,SRI
	26	L407B	Kafir/Durra	China,LNAAS		35	N133	Kaoliang/Caudatum	China,JLAAS
	27	TAM428B	Kafir/Durra	USA		36	J98H	Kaoliang/Caudatum	China,JLAAS
	28	N2B	Kafir/Durra	China,SXAU,SRI		37	JR108	Kaoliang/Caudatum	China,JLAAS
	29	J4190B	Kafir/Durra	China,JLAAS		38	XZ6936R	Kaoliang/Caudatum	China,SXAU,SRI
	30	SX3142B	Kafir/kuban	China,SXAU,SRI		39	9825R-1	Kaoliang/Caudatum	China,SXAU,SRI
	31	SJB	Kafir/Complex	China,SXAU,SRI		40	R111	Kaoliang/Caudatum	China,SXAU,SRI
	32	L45B	Kafir/Complex	China,SCAAS,SRI		41	HM65	Kaoliang/Caudatum	China,SXAU,SRI
	33	[Tx623B.Bmr6/L199B]B	Kafir/Complex	China,SXAU,SRI		42	LNH13	Kaoliang/Caudatum	China,SCAAS,SRI
	34	[L45B/(TX623B/V4B)]B	Kafir/Complex	China,SXAU,SRI		43	2381	Kaoliang/Caudatum	China,SXAU,SRI
	35	(SX605B×泸45B)B	Kafir/Complex	China,SXAU,SRI		44	LN8RN	Kaoliang/Caudatum	China,LNAAS
	36	7050B	Kafir/Complex	China,LNAAS		45	JR105	Kaoliang/Caudatum	China,JLAAS
	37	N4B	Kafir/Complex	China,SXAU,SRI		46	XLH-1	Kaoliang/Caudatum	China,HNAAS
	38	SX111B	Complex	China,SXAU,SRI		47	ZHOU	Kaoliang/Caudatum	China,LNAAS
**Type**	**Number**	**Name**	**Subspecies**	**Source**		48	1603N	Kaoliang/Caudatum	China,SXAU,SRI
**Restorer line**	1	Hegari	Caudatum	USA		49	5564F	Kaoliang/Caudatum	China,SXAU,SRI
	2	K35-Y5*1383	Complex	China,SXAU,SRI		50	5577F	Kaoliang/Caudatum	China,SXAU,SRI
	3	F-R	Durra	France		51	JR107	Kaoliang/Caudatum	China,JLAAS
	4	TX7078	Kafir	USA		52	zhzy2-07	Kaoliang/Caudatum	China,SXAU,SRI
	5	HTX430	Kafir	USA		53	363C/2691	Kaoliang/Complex	China,SXAU,SRI
	6	TX432	Kafir	USA		54	9198/TMS	Kaoliang/Complex	China,SXAU,SRI
	7	TX2737	Kafir	USA		55	IS7444C	Unknown	USA
	8	91633(H)	Kafir	Australia		56	XYLgaoliang	Unknown	Hungary
	9	91635(H)	Kafir	Australia		57	Feterita	Unknown	USA

The subspecies classification of the materials in Table 1 follows the five simple classification methods of Chinese breeders as mentioned in this paper. The abbreviations used for the material sources are as follows: China, SXAU, SRI (Sorghum Research Institute, Shanxi Agricultural University, China); China, JLAAS (Jilin Provincial Academy of Agricultural Sciences, China); China, LNAAS (Liaoning Provincial Academy of Agricultural Sciences, China); China, HBAAS (Hebei Provincial Academy of Agricultural Sciences, China); China, Farmer kind (Chinese farm sorghum varieties); China, CFAI (Chifeng Municipal Institute of Agricultural Science, China); China, XZAI (Xinzhou Agricultural Science Research Institute, China); China, SCAAS, SRI (Sorghum Research Institute, Sichuan Provincial Academy of Agricultural Sciences, China); China, HNAAS (Hunan Provincial Academy of Agricultural Sciences, China).

**Table 2:** PCR primer amplification sequence.

Primer Name	Primer Sequence
Dw1-F	TGGCGGTCCAACGTCTAAT
Dw1-R	CCTGAAGTATGGCGTGTCT
Dw_2_-F	CAGTTCAAATCAACGAGGAG
Dw_2_-R	TCCGTCGTGAAATGAGAATA
Dw_3_-F	CGTCATCGTCCAGAACTCGG
Dw_3_-R	GACCCTTGCTCCACCACCTT

The PCR primer amplification sequence source for Table 2 was obtained from the NCBI.

**Table 3:** Plant height gene types and measurement data of the test materials.

Plant Height Type	Genotype	Material Type	Number	Name	Subspecies	Plant Height (cm)	Stem Height (cm)	Spike Stalk Length (cm)	Spike Length (cm)	Height under Flag Leaf (cm)	Spike Stalk Length/Stem Height
Three-dwarf type	*Dw1Dw1* *dw2dw2* *dw3dw3* *dw4dw4*	Male sterile line	1	SX44B	Durra	115.00	88.67	39.00	26.33	49.67	0.44
			2	SX4244B	Durra	86.00	61.67	34.00	24.33	27.67	0.55
			3	A2V4B	Durra	126.33	101.33	37.33	25.00	64.00	0.37
		Restorer line	4	1602N	Kaoliang/Caudatum	115.00	88.00	30.67	27.00	57.33	0.35
		Avg				110.58	84.92	35.25	25.67	49.67	0.43
		SD				17.24	16.67	3.69	1.22	15.79	0.09
		COV				0.16	0.20	0.10	0.05	0.32	0.21
	*dw1dw1*	Male sterile line	5	2055B	Kafir/Durra	70.33	52.00	30.33	18.33	21.67	0.58
	*Dw2Dw2*		6	L407B	Kafir/Durra	103.00	76.00	41.67	27.00	34.33	0.55
	*dw3dw3*		7	TAM428B	Kafir/Durra	98.33	68.33	30.00	30.00	38.33	0.44
	*dw1dw1* *Dw2Dw2* *dw3dw3* *dw4dw4*		8	SJB	Kafir/Complex	126.33	100.33	44.67	26.00	55.67	0.45
			9	L45B	Kafir/Complex	144.33	107.67	44.67	36.67	63.00	0.41
			10	N2B	Kafir/Durra	107.00	72.33	39.00	34.67	33.33	0.54
			11	[Tx623B.Bmr6/L199B]B	Kafir/Complex	93.33	64.67	35.17	28.67	29.50	0.54
			12	TX3197B	Kafir	115.00	94.00	44.00	21.00	50.00	0.47
			13	[L45B/(TX623B/V4B)]B	Kafir/Complex	117.00	79.00	39.83	38.00	39.17	0.50
			14	(SX605B×L45B)B	Kafir/Complex	132.33	106.00	37.33	26.33	68.67	0.35
			15	(TXbmr6B/7501B)B	Kafir	108.67	80.67	42.00	28.00	38.67	0.52
			16	N1B	Kafir	103.00	69.00	39.00	34.00	30.00	0.57
			17	N3B	Kafir	116.33	84.00	50.17	32.33	33.83	0.60
			18	J4190B	Kafir/Durra	84.00	56.33	33.00	27.67	23.33	0.59
			19	ZSB	Kafir	127.67	103.67	48.33	24.00	55.33	0.47
			20	TX414B	Kafir	87.00	64.00	34.00	23.00	30.00	0.53
			21	TX639B	Kafir	132.50	108.33	49.00	24.17	59.33	0.45
			22	TX649B	Kafir	124.00	84.67	41.33	39.33	43.33	0.49
			23	TX623B	Kafir/Caudatum	128.00	96.00	52.00	32.00	44.00	0.54
			24	7501B	Kafir	104.33	84.00	43.50	20.33	40.50	0.52
			25	7050B	Kafir/Complex	128.50	95.50	37.00	33.00	58.50	0.39
			26	TX2925B	Kafir	113.00	87.67	43.67	25.33	44.00	0.50
			27	J16VII18B	Kafir	108.33	71.00	33.67	37.33	37.33	0.47
			28	J16VII27B	Kafir	102.67	73.33	36.00	29.33	37.33	0.49
			29	SX605B	Kafir	128.00	94.00	39.67	34.00	54.33	0.42
			30	SX77B	Kafir	104.00	70.33	44.33	33.67	26.00	0.63
			31	CS3541B	Durra	111.33	82.33	50.67	29.00	31.67	0.62
			32	SX3142B	Kafir/kuban	89.67	65.67	26.33	24.00	39.33	0.40
			33	(314B/623B)B	Kafir/Caudatum	112.67	88.00	39.67	24.67	48.33	0.45
		Restorer line	34	LNR	Kaoliang/Caudatum	131.67	108.67	20.67	23.00	88.00	0.19
			35	XLH*GN2	Kaoliang/Caudatum	104.00	78.67	29.00	25.33	49.67	0.37
			36	0592F	Kaoliang/Caudatum	92.17	63.17	37.10	29.00	26.07	0.59
			37	TX7078	Kafir	112.67	89.67	46.67	23.00	43.00	0.52
			38	HTX430	Kafir	108.00	74.00	44.25	34.00	29.75	0.60
			39	TX432	Kafir	91.00	67.00	44.00	24.00	23.00	0.66
			40	TX414	Kafir/Durra	87.33	66.67	36.67	20.67	30.00	0.55
			41	TX2737	Kafir	69.83	46.00	26.67	23.83	19.33	0.58
			42	91633(H)	Kafir	97.67	74.33	37.83	23.33	36.50	0.51
			43	91635(H)	Kafir	91.00	69.00	39.00	22.00	30.00	0.57
			44	91644(H)	Kafir	103.50	74.50	46.00	29.00	28.50	0.62
			45	91645(H)	Kafir	105.67	77.67	43.33	28.00	34.33	0.56
			46	91648	Kafir	94.33	70.33	42.33	24.00	28.00	0.60
			47	SX1042	Kaoliang/Kafir	107.00	74.33	38.67	32.67	35.67	0.52
			48	961547	Kafir	95.00	73.33	42.33	21.67	31.00	0.58
			49	91624(H)	Kafir	92.50	63.07	39.17	29.43	23.90	0.62
			50	SX861	Kaoliang/Caudatum	97.67	68.67	19.33	29.00	49.33	0.28
		Avg				106.56	78.65	39.20	27.91	39.45	0.51
		SD				16.47	15.14	7.34	5.22	13.88	0.10
		COV				0.15	0.19	0.19	0.19	0.35	0.19
	*dw1dw1,dw2dw2* *Dw3Dw3,dw4dw4*	Restorer line	51	NJ426	Kafir/Durra	90.33	62.67	39.00	27.67	23.67	0.62
Two-dwarf type	*dw1dwd1,Dw2Dw2* *Dw3Dw3,dw4dw4*	Male sterile line	52	SX111B	Complex	97.33	74.67	37.50	22.67	37.17	0.50
			53	998B	Kafir	117.67	91.67	49.17	26.00	42.50	0.54
			54	961542B	Durra	95.67	69.00	47.00	26.67	22.00	0.68
		Avg				103.56	78.45	44.56	25.11	33.89	0.57
		SD				12.25	11.80	6.21	2.14	10.64	0.09
		COV				0.12	0.15	0.14	0.09	0.31	0.16
	*Dw1Dw1,dw2dw2* *Dw3Dw3,dw4dw4*	Restorer line	55	Hegari	Caudatum	217.00	195.00	53.00	22.00	142.00	0.27
	*Dw1Dw1* *Dw2Dw2* *dw3dw3* *dw4dw4*	Male sterile line	56	TV33B	Kafir	78.00	66.00	28.67	12.00	37.33	0.43
			57	N4B	Kafir/Complex	143.67	102.67	43.67	41.00	59.00	0.43
			58	F4B	Durra	153.50	126.00	32.50	27.50	93.50	0.26
		Restorer line	59	HC356	Kaoliang/Caudatum	157.00	129.00	33.33	28.00	95.67	0.26
			60	1383-2	Kaoliang/Caudatum	133.67	108.00	21.00	25.67	87.00	0.19
			61	zhzy2-07	Kaoliang/Caudatum	116.00	95.00	23.00	21.00	72.00	0.24
			62	N133	Kaoliang/Caudatum	118.00	99.00	26.50	19.00	72.50	0.27
			63	363C/2691	Kaoliang/Caudatum	136.33	117.67	27.67	18.67	90.00	0.24
			64	J98H	Kaoliang/Caudatum	126.00	91.33	28.33	34.67	63.00	0.31
			65	F-R	Durra	104.00	80.33	42.67	23.67	37.67	0.53
			66	JR108	Kaoliang/Caudatum	130.67	99.33	39.33	31.33	60.00	0.40
			67	X6936R	Kaoliang/Caudatum	95.00	66.00	40.00	29.00	26.00	0.61
			68	9198/TMS	Kaoliang/Caudatum	110.00	74.00	33.67	36.00	40.33	0.46
			69	IS7444C	Unknown	129.00	111.67	51.60	17.33	60.07	0.46
			70	HBNR436-2	Kaoliang/Kafir	105.50	73.50	26.50	32.00	47.00	0.36
			71	9825R-1	Kaoliang/Caudatum	93.67	66.67	31.33	27.00	35.33	0.47
			72	R111	Kaoliang/Caudatum	137.50	113.00	26.00	24.50	87.00	0.23
			73	XYLgaoliang	Unknown	137.67	110.33	32.33	27.33	78.00	0.29
			74	HM65	Kaoliang/Caudatum	161.33	136.33	37.00	25.00	99.33	0.27
			75	LNH13	Kaoliang/Caudatum	133.33	103.67	24.00	29.67	79.67	0.23
			76	2381	Kaoliang/Caudatum	134.00	110.00	26.50	24.00	83.50	0.24
			77	XL7	Kaoliang/Caudatum	142.33	118.67	34.33	23.67	84.33	0.29
			78	Jing liang 5	Kaoliang/Caudatum	138.00	109.73	26.83	28.27	82.90	0.24
			79	SXR0-30	Kaoliang/Caudatum	124.67	101.33	33.33	23.33	68.00	0.33
			80	ZHOU	Kaoliang/Caudatum	111.00	85.67	28.67	25.33	57.00	0.33
			81	JR105	Kaoliang/Caudatum	124.67	93.67	37.00	31.00	56.67	0.40
			82	1603N	Kaoliang/Caudatum	113.67	85.33	27.00	28.33	58.33	0.32
			83	0-30/DHS	Kaoliang/Caudatum	136.67	112.00	35.00	24.67	77.00	0.31
			84	5564F	Kaoliang/Caudatum	94.33	69.00	26.67	25.33	42.33	0.39
			85	5577F	Kaoliang/Caudatum	100.67	73.67	29.67	27.00	44.00	0.40
		Avg				124.00	97.62	31.80	26.38	65.82	0.34
		SD				20.28	19.97	6.84	5.74	20.55	0.10
		COV				0.16	0.20	0.21	0.22	0.31	0.30
One-dwarf type	*Dw1Dw1* *Dw2Dw2* *Dw3Dw3* *dw4dw4*	Restorer line	86	LN8RN	Kaoliang/Caudatum	128.33	106.33	28.67	22.00	77.67	0.27
			87	XLH-1	Kaoliang/Caudatum	179.00	153.67	35.00	25.33	118.67	0.23
			88	Hong yin zi	Kaoliang	247.33	228.00	48.33	19.33	179.67	0.21
			89	Feterita	Unknown	215.00	188.50	31.83	26.50	156.67	0.17
			90	QKY	Kaoliang	291.00	270.67	35.00	20.33	235.67	0.13
			91	K35-Y5*1383	Complex	101.33	78.00	21.67	23.33	56.33	0.28
			92	JR107	Kaoliang/Caudatum	120.67	87.33	35.50	33.33	51.83	0.41
			93	chf5933	Kaoliang	153.67	121.00	35.33	32.67	85.67	0.29
			94	SCS	Kaoliang	138.00	109.73	26.83	28.27	82.90	0.24
			95	20131937	Kaoliang	221.00	205.33	32.27	15.67	173.07	0.16
		Avg				179.53	154.86	33.04	24.68	121.82	0.24
		SD				62.00	65.36	7.01	5.71	61.57	0.08
		COV				0.35	0.42	0.21	0.23	0.51	0.34

Table 3 is categorized based on the plant height genotype of the material, and the provided measurements represent the averages of three replicates. In the table, “Avg” denotes the average of the data, “SD” represents the standard deviation, and “COV” indicates the coefficient of variation. All data values have been retained up to two decimal places.

**Table 4:** Sorghum sterile line material diallel hybrid F_1_ strain height results.

Male Sterile Line A/B and Genotype		A2V4B	SX44B	CS3541B	TX623B	TX3197B
		(Durra)	(Durra)	(Durra)	(Kafir/Caudatum)	(Kafir)
		*Dw1-dw2-dw3-dw4*	*Dw1-dw2-dw3-dw4*	*dw1-Dw2-dw3-dw4*	*dw1-Dw2-dw3-dw4*	*dw1-Dw2-dw3-dw4*
A2V4A (Durra)	F_1_ genotype		*Dw1Dw1-dw2dw2-dw3dw3-dw4dw4*	*Dw1dw1-Dw2dw2-dw3dw3-dw4dw4*	*Dw1dw1-Dw2dw2-dw3dw3-dw4dw4*	*Dw1dw1-Dw2dw2-dw3dw3-dw4dw4*
*Dw1-dw2-dw3-dw4*	Plant height (cm)	126.33	132.60	220.11	206.67	181.00
	Stem height (cm)	101.33	108.25	189.33	178.01	147.67
	Spike stalk length (cm)	37.33	38.54	72.33	52.67	50.33
	Spike stalk length/stem height	0.37	0.36	0.38	0.30	0.34
SX44A (Durra)	F_1_ genotype	*Dw1Dw1-dw2dw2-dw3dw3-dw4dw4*		*Dw1dw1-Dw2dw2-dw3dw3-dw4dw4*	*Dw1dw1-Dw2dw2-dw3dw3-dw4dw4*	*Dw1dw1-Dw2dw2-dw3dw3-dw4dw4*
*Dw1-dw2-dw3-dw4*	Plant height (cm)	130.26	115.12	175.22	177.21	187.55
	Stem height (cm)	102.53	90.21	149.52	151.25	161.32
	Spike stalk length (cm)	37.25	32.14	50.12	52.26	57.35
	Spike stalk length/stem height	0.36	0.36	0.34	0.35	0.36
CS3541A (Durra)	F_1_ genotype	*Dw1dw1-Dw2dw2-dw3dw3-dw4dw4*	*Dw1dw1-Dw2dw2-dw3dw3-dw4dw4*		*dw1dw1-Dw2DW2-dw3dw3-dw4dw4*	*dw1dw1-Dw2DW2-dw3dw3-dw4dw4*
*dw1-Dw2-dw3-dw4*	Plant height (cm)	217.00	177.32	111.33	164.33	106.33
	Stem height (cm)	176.67	150.23	82.33	133.00	83.67
	Spike stalk length (cm)	67.00	52.12	50.67	66.01	43.67
	Spike stalk length/stem height	0.38	0.35	0.62	0.50	0.52
TX623A (Kafir/Caudatum)	F_1_ genotype	*Dw1dw1-Dw2dw2-dw3dw3-dw4dw4*	*Dw1dw1-Dw2dw2-dw3dw3-dw4dw4*	*dw1dw1-Dw2DW2-dw3dw3-dw4dw4*		*dw1dw1-Dw2DW2-dw3dw3-dw4dw4*
*dw1-Dw2-dw3-dw4*	Plant height (cm)	200.01	182.21	154.67	128.12	140.33
	Stem height (cm)	167.67	150.23	124.67	96.01	114.33
	Spike stalk length (cm)	54.67	49.52	67.67	52.00	40.01
	Spike stalk length/stem height	0.33	0.33	0.54	0.54	0.35
TX3197A (Kafir)	F_1_ genotype	*Dw1dw1-Dw2dw2-dw3dw3-dw4dw4*	*Dw1dw1-Dw2dw2-dw3dw3-dw4dw4*	*dw1dw1-Dw2DW2-dw3dw3-dw4dw4*	*dw1dw1-Dw2DW2-dw3dw3-dw4dw4*	
*dw1-Dw2-dw3-dw4*	Plant height (cm)	175.12	168.41	107.67	143.67	115.01
	Stem height (cm)	147.67	140.12	83.67	115.01	94.00
	Spike stalk length (cm)	46.33	44.21	40.10	48.00	44.00
	Spike stalk length/stem height	0.31	0.32	0.48	0.42	0.47

The values presented in Table 4 represent the average plant height measurements from three replicates of the hybrid F1 generation.

**Table 5:** Plant height genotypes in sterile and recovered lines tested in F_1_ generation.

Hybridized Combination Genotype		Hybridized Combination				Average (cm)
*Dw1dw1-Dw2dw2-dw3dw3-dw4dw4*	Hybridized combination	A2V4A × TX7078	A2V4A × SX1042	SX44A × TX7078	SX44A × SX1042	
	Plant height (cm)	192.33	193.5	172.58	182.53	185.24
*Dw1Dw1-Dw2dw2-dw3dw3-dw4dw4*	Hybridized combination	A2V4A × Jing liang 5	A2V4A × SXR0-30	SX44A × Jing liang 5	SX44A × SXR0-30	
	Plant height (cm)	232.4	220.67	202.15	175.21	207.61
*dw1dw1-Dw2Dw2-dw3dw3-dw4dw4*	Hybridized combination	TX623A × TX7078	TX623A × SX1042	TX3197A × TX7078	TX3197A × SX1042	
	Plant height (cm)	133.33	135.22	110	127.33	126.47
*Dw1dw1-Dw2Dw2-dw3dw3-dw4dw4*	Hybridized combination	TX623A × Jing liang 5	TX623A × SXR0-30	TX3197A × Jing liang 5	TX3197A × SXR0-30	
	Plant height (cm)	203.67	194.33	195.33	184	194.33

Table 5 shows the mean values and genotypes of plant height measurements in F_1_ generation crosses. The term “average” represents the mean value of the plant height data for each respective genotype.

## Data Availability

Data are contained within the article and Supplementary Materials.

## Supplementary Materials

The following supporting information can be downloaded at: https://www.mdpi.com/article/10.3390/genes15010083/s1, Figure S1: Sequence alignment of *Dw1* gene of the tested materials. Sequencing sample ID (”R” is Restorer line number, “B” is Male sterile line number) were listed on the left of the picture, and the sequence of the samples were performed on the right side of the picture. An alternative of “A” to “T” at position 178 of the sequence led the genotype changed from *Dw1Dw1* to *dw1dw1*; Figure S2: Sequence alignment of *Dw2* gene of the tested materials. Sequencing sample ID (“R” is Restorer line number, “B” is Male sterile line number) were listed on the left of the picnture, and the sequence of the samples were performed on the right side of the picture. A deletion of “T” at position 11 of the sequence led the genotype changed from *Dw2Dw2* to *dw2dw2*; Figure S3: Genotype characterization of *Dw3* gene in tested materials. ID of materials were numbered as “R” and “B”, and the target band size of *Dw3Dw3* genotype is 1263 bp, and the target band size of *dw3dw3* genotype is 2154 bp. Size marker: DL2000. PCR products were saparated in 1% agarose gel.
