# Supplementary material for: Research on Genotype Markers for Plant Height and Assisted Breeding of Key Sorghum Resources in China
Source: Genes (Basel). 2024 Jan 9;15(1):83. doi: 10.3390/genes15010083 (PMC10815169; doi:10.3390/genes15010083)
Supplement: Supplementary file 1 [file genes-15-00083-s001.zip › genes-2781553-supplementary.pdf]

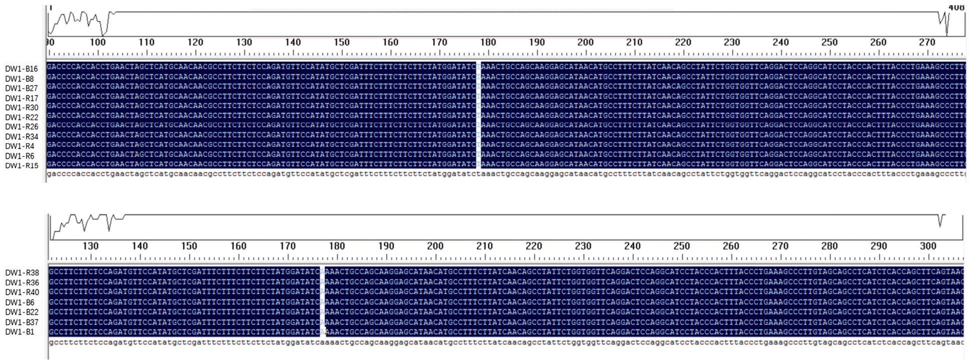

Supplemental Figure S1. Sequence alignment of *Dw1* gene of the tested materials. Sequencing sample ID ("R" is Restorer line number, "B" is Male sterile line number) were listed on the left of the picture, and the sequence of the samples were performed on the right side of the picture. An alternative of "A" to "T" at position 178 of the sequence led the genotype changed from *Dw1Dw1* to *dw1dw1*.

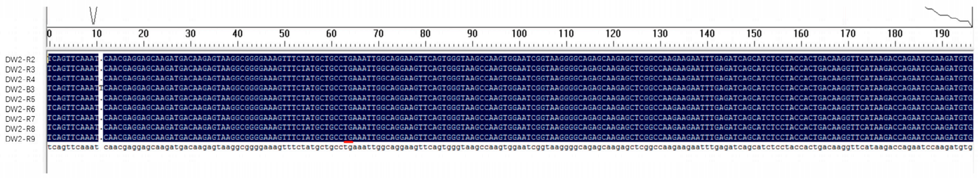

Supplemental Figure S2. Sequence alignment of *Dw2* gene of the tested materials. Sequencing sample ID ("R" is Restorer line number, "B" is Male sterile line number) were listed on the left of the picnture, and the sequence of the samples were performed on the right side of the picture. A deletion of "T" at position 11 of the sequence led the genotype changed from *Dw2Dw2* to *dw2dw2*.

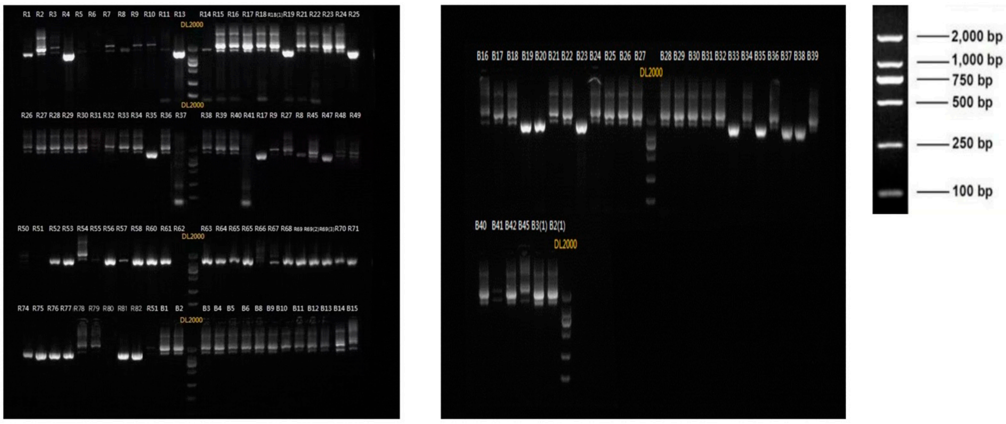

Supplemental Figure S3. Genotype characterization of *Dw3* gene in tested materials. ID of materials were numbered as “R” and “B”, and the target band size of *Dw3Dw3* genotype is 1263 bp, and the target band size of *dw3dw3* genotype is 2154 bp. Size marker: DL2000. PCR products were saparated in 1% agarose gel.
